# Rotation Alleviated the Continuous Cropping Obstacle of Peanut (*Arachis hypogaea* L.) Cultivation and Optimized the Endophytic Bacterial Communities in Peanut Pods

**DOI:** 10.3390/plants14121799

**Published:** 2025-06-12

**Authors:** Miao Liu, Pu Shen, Qi Wu, Haiyan Liang, Dianxu Chen, Liyu Yang

**Affiliations:** Shandong Peanut Research Institute/Key Laboratory of Peanut Biology, Genetics & Breeding, Ministry of Agriculture and Rural Affairs, Shandong Academy of Agricultural Sciences, 126 Wannianquan Road, Qingdao 266100, China; liumiao123123@163.com (M.L.); shenpupeanut@126.com (P.S.); qi_wu@126.com (Q.W.); 15094073735@163.com (H.L.); chenpeanut@126.com (D.C.)

**Keywords:** *Arachis hypogaea* L., crop rotation, green manure, peanut pod, endophytic bacteria

## Abstract

Peanut (*Arachis hypogaea* L.) continuous cropping reduces yield and quality, but the effects of specific rotation methods on yield and endophytic bacterial colonization remain unclear. Based on five years of continuous cropping trial data, three different cropping systems (WF, annual summer peanut and winter fallow; GM, annual summer peanut and winter ryegrass; CR, summer peanut rotated with summer maize and winter wheat) were employed in this study to systematically analyze and evaluate the effectiveness of crop rotation in mitigating peanut continuous cropping obstacles and its underlying mechanisms. The results showed that CR increased pod yield by 33% and kernel nitrogen content by 6.8% compared to WF, while GM had a marginal effect (1.4% nitrogen increase). Microbial analysis (LEfSe/LDA) revealed that CR enriched beneficial bacteria (e.g., *Actinobacteria*, *Corynebacteriales*) in pods while suppressing potential pathogens (e.g., *Gammaproteobacteria*, *Burkholderiales*). These findings demonstrate that strategic crop rotation, particularly CR, mitigates continuous cropping obstacles by enhancing yield, improving kernel quality, and promoting beneficial endophytic bacterial communities. Our findings highlight the complexity of crop rotation system functioning and how interactions between cropping patterns and endophytic microbiota affect peanut yield and kernel quality.

## 1. Introduction

Peanuts (*Arachis hypogaea* L.) rank among the world’s most important oil crops; they are valued for their high oil content and nutritional benefits, and they serve as a key edible oil source globally [1]. However, limited suitable cultivation areas force farmers to practice continuous peanut cropping, which triggers serious agricultural challenges, known as continuous cropping obstacles [2]. These obstacles manifest as declining soil fertility, escalating pest and disease pressures, and deteriorating soil health; they restrict the sustainable development of the peanut industry and cause major economic losses to global agricultural production [3,4]. Continuous cropping reduces beneficial microbes but increases harmful ones, disrupting soil microbial balance [5]. This impairs nutrient cycling and peanut growth, while accumulated secondary metabolites further inhibit development [6,7,8,9].

Selecting appropriate cropping systems is crucial for alleviating the continuous cropping obstacles of peanuts. Crop rotation is an effective approach to address this issue [10]. By rotating other crops, such as maize, soybeans, or wheat, the life cycle of soil-borne pathogens and pests can be effectively disrupted, thereby mitigating their detrimental effects on peanut productivity [11,12]. Furthermore, rotational cultivation modulates soil nutrient dynamics and sustains soil fertility [13]. Zou et al. (2021) have demonstrated that maize–peanut rotation systems significantly enhanced soil microbial biomass and community diversity, improved soil carbon pool characteristics, and increased nutrient bioavailability relative to monocropped peanut systems [14]. A comparative analysis revealed that rotational cropping reduced the heterogeneity of rhizosphere microbial composition in peanut cultivation systems, inducing a more uniform assembly of root-associated microbial communities. This microbial community restructuring optimizes the soil micro-ecosystem in long-term peanut cultivation systems [15]. Therefore, crop rotation is very important for alleviating the imbalance of the soil community structure in peanut continuous cropping and improving peanut growth, yield, and quality.

Plant endophytes are microorganisms, including bacteria, fungi, and actinomycetes, that colonize the interior of plant tissues (e.g., roots, stems, leaves, seeds, etc.) without causing obvious diseases. They form a mutually beneficial symbiotic relationship with the host plant and play a key role in promoting plant growth, enhancing disease resistance, and improving stress tolerance [16,17,18]. Endophytes usually enter plant tissues through stomata, lenticels, lateral roots, root hairs, and natural wounds during plant growth, and this specific colonization pathway has led to the establishment of a close ecological association between soil microorganisms and plant endophyte communities. Based on this close ecological association, the composition and function of endophyte communities showed a significant correlation with the cropping pattern system [19,20]; however, the effects of different cropping patterns on the diversity, abundance, and ecological function of plant endophyte communities have not been fully analyzed. Peanut is an important underground fruiting crop, and its pod development is directly affected by the soil microbial environment. Compared to the aboveground part, the endophyte community of peanut pods may have a unique composition and function and play a key role in nutrient uptake, disease resistance, and pod expansion. However, there is still a lack of research on endophytes in peanut pods, especially under different cropping patterns; the pattern of change in the structure and function of pod endophyte communities and their effects on pod development is still not clear.

**Figure 1:**
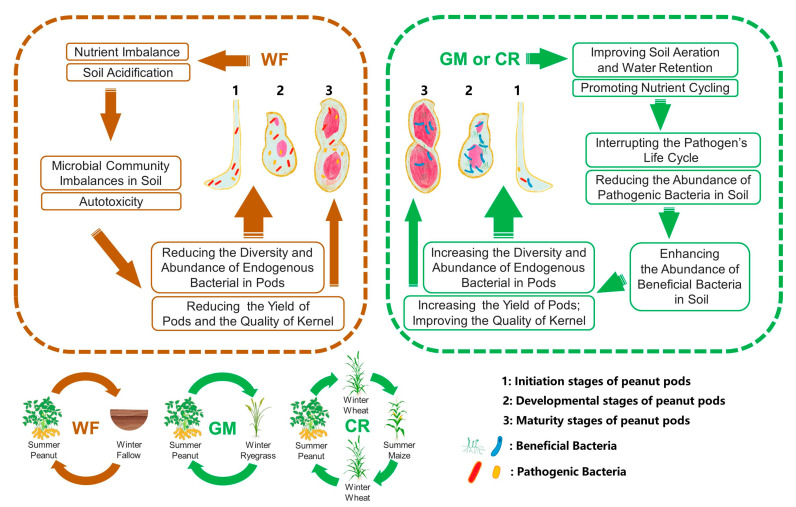
Concept diagram of the effects of different cropping systems on pod yield and endophytic bacterial community in peanut (*Arachis hypogaea* L.) pods.

Peanut is an important oilseed crop globally, and its yield is severely constrained by continuous crop failure. Although previous studies have shown that different cropping patterns can alleviate peanut crop failure through soil microbial restructuring, their specific effects on the structure and function of endophyte communities in peanut pods are still poorly understood. The main objective of this study was to assess the effects of different cropping patterns on peanut pod nutrient content, yield, and endophytic bacterial community structure and function, as well as to explore the potential mechanisms by which crop rotation-induced changes in endophytic bacteria are associated with increased productivity. We hypothesized that under continuous cropping conditions, changes in endophytic bacteria may directly affect the nutritional and developmental status of peanut pods, resulting in lower yield and quality. By employing different crop rotation patterns, different functional characteristics of endophytic bacteria in pods will occur under different cropping systems, and different rotation patterns will increase the quantity and diversity of beneficial endophytic microorganisms, thereby improving pod development (Figure 1). By integrating agronomic and microbiome data, this work provides new mechanistic insights into how rotation systems optimize peanut production through alterations in peanut pod endophytic microbes, offering science-based strategies for sustainable peanut cultivation.

## 2. Materials and Methods

### 2.1. Study Site Description and Experimental Design

This five-year localization experiment was conducted at a typically acidic brown soil (Haplic Luvisols, the FAO soil classification system) in a major peanut production area, Laixi city, Shandong (E 120°29′, N 36°48′, 227 m above sea level), China. At the experimental site, peanut crops had never been planted in the brown soil. The average annual temperature was 11.5 °C, and the average annual rainfall was 635.8 mm.

In a randomly complete block design with three replicates for each treatment, three continuously experimental treatments were examined: (1) WF, annual summer peanut (cv. Huayu 33) and winter fallow; (2) GM, annual summer peanut (cv. Huayu 33) and winter ryegrass (cv. Dongmu 70) as green manure; (3) CR, summer peanut (cv. Huayu 33) rotated with summer maize (cv. Zhengdan 958) and winter wheat (cv. Luyuan 502). All crops were grown under conventional cultivation practices. Nitrogen (N), phosphorus (P), and potassium (K) fertilizers were based on the local fertilization practice. A total of 750 kg/hm^2^ ternary compound fertilizer (15% N: 15% P_2_O_5_: 15% K_2_O) was applied containing 112.5 kg N/hm^2^, 112.5 kg P_2_O_5_/hm^2^, and 112.5 kg K_2_O/hm^2^.

### 2.2. Determination of Major Elements in Peanut Kernels

For nitrogen determination, fresh plant tissues were dried at 65 °C to constant weight in an air oven, then dried samples were finely ground using a stainless steel mill to pass through a 100-mesh sieve, with 0.5–1.0 g aliquots digested in concentrated H_2_SO_4_ (10 mL) with a K_2_SO_4_-CuSO_4_ catalyst (10:1 ratio) at 420 °C for 2 h until clear; the cooled digest was then distilled with 40% NaOH to liberate NH_3_, which was trapped in 4% boric acid containing mixed indicator and titrated with standardized 0.1 N HCl to determine nitrogen content [21]. For phosphorus determination, fresh plant tissues were oven-dried at 65 °C to constant weight and ground to a fine powder (100-mesh), then the samples were digested by sulfuric acid–hydrogen peroxide (5 mL concentrated H_2_SO_4_ + 2 mL 30% H_2_O_2_) at 350 °C for 1 h. Digested samples were analyzed by the molybdenum blue method, where a 2 mL aliquot was mixed with ammonium molybdate–ascorbic acid reagent, and absorbance was measured at 880 nm after 30 min of color development [22]. For the determination of potassium and calcium, samples underwent microwave-assisted digestion (5 mL concentrated HNO_3_ + 1 mL H_2_O_2_) at 180 °C for 15 min. The digest was diluted to 50 mL with deionized water before analysis by flame atomic absorption spectrometry (air-acetylene flame, 10 cm slot burner) with K measured at 766.5 nm (red filter) and Ca at 422.7 nm (nitrous oxide-acetylene flame, with 1% LaCl_3_ added to suppress interference) [23].

### 2.3. DNA Extraction, Amplification, and Sequencing of Pod Samples

Before DNA extraction, all peanut pod samples were cleaned with deionized water, followed by surface disinfection with 75% ethanol and 2.5% sodium hypochlorite. After disinfection, the residual ethanol and sodium hypochlorite were rinsed off with deionized water. The total genomic DNA of 27 pod tissue samples was extracted separately using the Fast DNA^®^ Spin Kit for Plant Tissue (MP Biomedicals, Thomas Irvine, CA, USA) according to the manufacturer’s instructions. The concentration and purity of DNA were then examined with 1% agarose gels. The V3-V4 region of the bacterial gene was amplified using primers 515F (5′-GTGCCAGCMGCCGCGG-3′) and 806R (5′-GGACTACHVGGGTWTCTAAT-3′). All PCR reactions were conducted using a TransGen Kit (TransGen AP221-02: TransStart Fastpfu DNA Polymerase, TransGen Biotech, Beijing, China) with a 20 μL reaction system, including 4 μL FastPluBuffer, 0.4 μL FastPfu Polymerase, 2 μL dNTPs, 0.8 μL of each forward and reverse primers, 0.2 μL Bovine Serum Albumin, and 10 ng template DNA. DNA was amplified using ABI GeneAmp^®^ 9700 (Life Technologies, Foster City, CA, USA) under the following conditions: 95 °C for 5 min, followed by 95 °C for 3 min, 30 cycles of 95 °C for 30 s, 55 °C for 30 s, 72 °C for 45 s, and elongation at 72 °C for 10 min. After purification, all PCR products were used to construct sequencing libraries by using the TruSeq^TM^ DNA Sample Prep Kit (Illumina, San Diego, CA, USA). Ultimately, the constructed libraries were sequenced on an Illumina HiSeq2500 platform at Majorbio Bio-Pharm Technology (Shanghai, China).

### 2.4. Statistical Analysis and Bioinformatics Analysis

Statistical analyses performed in this study were considered significant at *p* < 0.05. All the experimental data in the study were analyzed using SPSS version 23 (SPSS, Inc., Chicago, IL, USA). The application of different cropping systems in the macronutrient content of peanut kernels and peanut yield was compared by using one-way ANOVA, and then the differences were detected by the honestly significant difference test (Tukey-HSD, *p* < 0.05). Figures were constructed using the software Prism Graphpad Prism 8.0. Alpha diversity (Shannon and Ace indices) was analyzed using Mothur (v1.30.2). Principal coordinate analysis (PCoA) was performed in R (v3.3.1) to compare the similarity or dissimilarity of microbial community composition among different groups. Partial least squares discriminant analysis (PLS-DA) was conducted using the mixOmics package in R (v3.3.1) to assess sample similarity across treatments and evaluate the significance of inter-group differences. Kruskal–Wallis rank-sum tests were applied using the stats package in R (v3.3.1) and the scipy package (v1.0.0) in Python to identify significant differences in microbial taxa among samples. Linear discriminant analysis (LDA) Effect Size (LEfSe) (http://huttenhower.sph.harvard.edu/LEfSe (accessed on 3 December 2024)) was employed to identify microbial taxa that contributed significantly to differences between treatment groups based on taxonomic composition. To predict the functional potential of the pod-associated microbiota, PICRUSt software (v2.2.0) was used to annotate COG families and KEGG Orthologs (KOs), followed by functional pathway and abundance profiling. Finally, Redundancy Analysis (RDA) and Spearman correlation heatmaps were generated using the vegan package (v2.4.3) in R to explore the relationships between endophytic bacterial communities and the macroelement content of peanut kernels, as well as peanut yield. All bioinformatics analyses were completed on the I-sanger platform (Majorbio Bio-Pharm Technology Co., Ltd., Shanghai, China). Data in the figures were reported as means + SD for three replicate analyses.

## 3. Results

### 3.1. Differences in the Macronutrient Content of Peanut Kernels and Peanut Yield Were Observed Under Various Planting Modes

After five consecutive years of planting peanuts using the same cropping systems, a comprehensive examination of peanut kernel macronutrient contents and peanut yields was conducted. The results revealed that the three cultivation modes led to differences in the macronutrient contents of peanut kernels. Specifically, peanuts under crop rotation (CR) had a higher nitrogen content than those under green manure (GM) and winter fallow (WF) systems; no significant differences in peanut nitrogen content were found between GM and WF. No significant differences in phosphorus content were observed among the three planting modes. However, WF peanuts had the highest potassium content, followed by GM peanuts, while CR peanuts had the lowest. Similarly, WF peanuts had the highest calcium content, followed by GM peanuts, with CR peanuts having the lowest calcium content. In terms of peanut yield, CR had the highest yield, while GM and WF had lower yields with no significant difference between them, consistent with the nitrogen content in peanut kernels (Table 1).

### 3.2. The α-Diversity Analysis of Endophytic Bacteria in Peanut Pods Under Different Planting Patterns

**Figure 2:**
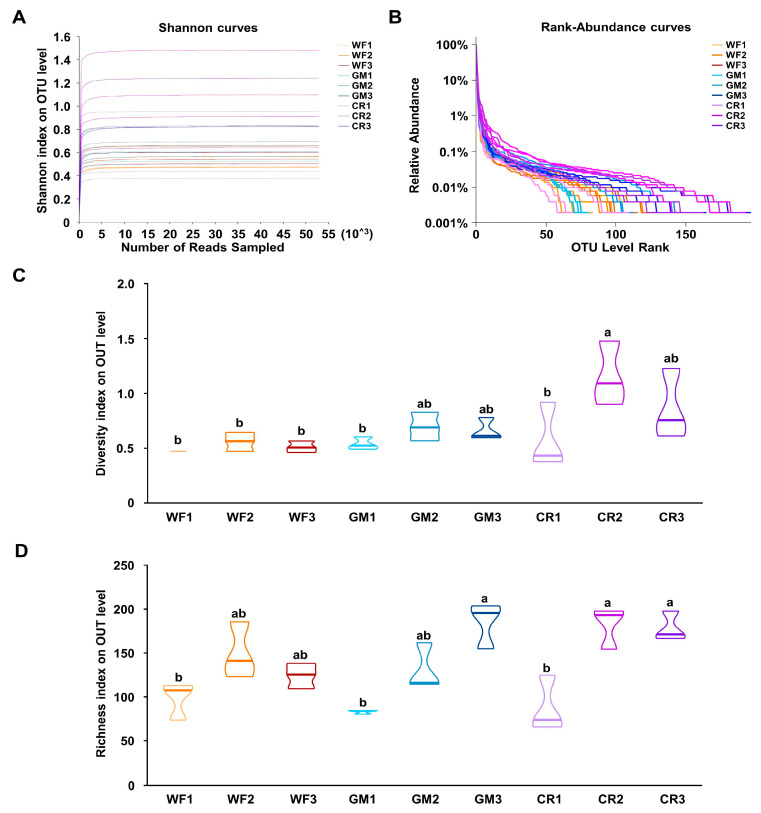
α diversity analysis of endophytic bacterial communities on the peanut pod. (**A**) Rarefaction curve analysis. (**B**) Rank abundance curve. (**C**) Diversity index tested by Shannon on OUT level. (**D**) Richness index tested by Ace on OUT level. 1: Initiation stages of pods; 2: developmental stages of pods; 3: maturity stages of pods. Different lowercase letters above bars indicate significant differences (*p* < 0.05; one-way ANOVA, followed by Tukey’s HSD test). The same below.

α-Diversity analysis can estimate the richness and diversity of bacterial communities. Among them, the rarefaction curve is an effective tool that can assess whether the sequencing depth is sufficient to cover all taxa and indirectly reflect the species richness in the sample. As shown in Figure 2A, when the sequencing depth reached 10,000, the curves for all samples gradually leveled off, demonstrating that the sequencing depth was adequate to cover most of the species within the samples. Moreover, the rank–abundance curve provides a visual representation of the evenness and richness of species in each sample. As shown in Figure 2B, when the OTU count exceeded 100, the curve became relatively flat, indicating a more even distribution of species. These results provide us with a deeper understanding of the structure and composition of bacterial communities.

The analysis of bacterial α-diversity indices at the OTU level for each peanut pod sample revealed the following findings (Figure 2C): during the initiation stages, there were no significant differences in Shannon indices among the three cropping systems, and the diversity index was relatively low; during the developmental stages, both GM and CR showed a significant increase in diversity index compared to WF, with CR having the highest value; in the maturity stages, the situation was similar to that in the developmental stages, with CR having the highest diversity index, followed by GM and then WF. Richness, analyzed using the Chao index (Figure 2D), indicated that during the initiation stages, all three cropping systems had low diversity indices without significant differences; in the developmental stages, the diversity indices of all three cropping systems increased, with CR having the highest value, while in the maturity stages, the diversity indices of the three cropping systems were comparable to those in the developmental stages.

### 3.3. Circus Diagram Showing the Dominant Flora of Different Samples at Different Species Classification Levels

**Figure 3:**
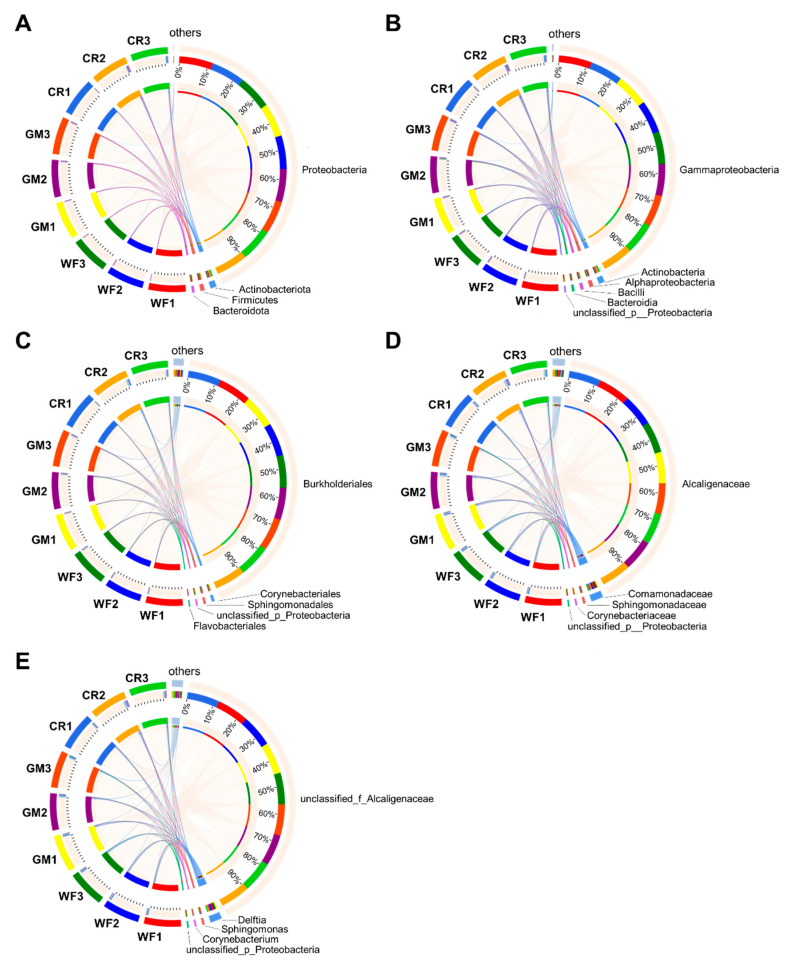
Circus plots visualize microbial community composition at phylum (**A**), class (**B**), order (**C**), family (**D**), and genus (**E**) levels, showing the relative abundance and distribution of dominant taxa across samples. Each circular diagram is divided into colored segments representing taxonomic units, with segment size proportional to their relative abundance in each sample. The percentage labels indicate the proportion of each taxon within the community.

At the phylum level (Figure 3A), the dominant phyla in all samples were Actinobacteria, Firmicutes, and Bacteroidota. At the class level (Figure 3B), the dominant classes in all peanut kernel samples were Gammaproteobacteria, Actinobacteria, Alphaproteobacteria, unclassified_p_Proteobacteria, Bacteroidia, and Bacilli. At the order level (Figure 3C), the shared dominant orders among all peanut kernel samples were Corynebacteriales, Sphingomonadales, unclassified_p_Proteobacteria, and Flavobacteriales. At the family level (Figure 3D), the dominant families in all peanut pod samples were Comamonadaceae, Sphingomonadaceae, Corynebacteriaceae, and unclassified_p_Proteobacteria. At the genus level (Figure 3E), the shared dominant genera among all peanut kernel samples were *Delftia*, *Sphingomonas*, *Corynebacterium*, and *unclassified_p_Proteobacteria*.

### 3.4. β-Diversity Analysis of Endophytic Bacteria in Peanut Pods Under Different Planting Patterns

**Figure 4:**
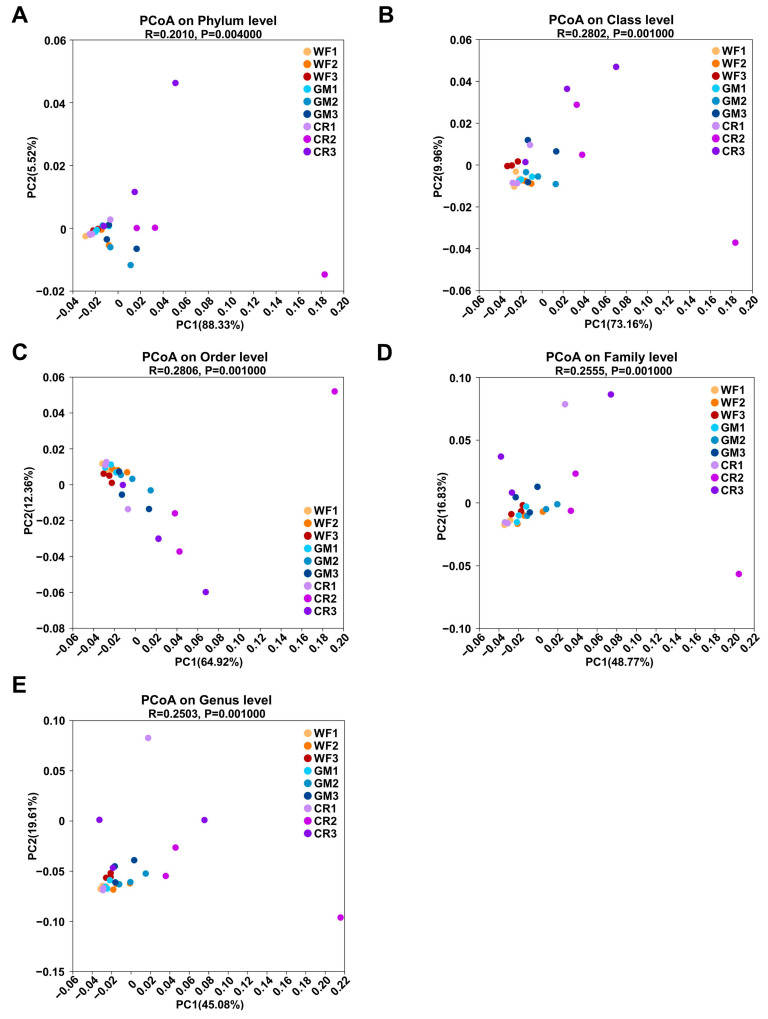
PCoA plots visualize β diversity of microbial communities across phylum (**A**), class (**B**), order (**C**), family (**D**), and genus (**E**) levels. Scatter plots display sample distribution based on principal coordinates (PC1 and PC2), with percentages indicating variance explained by each axis. Different colors/symbols represent experimental groups. R and *p* values on each plot reflect statistical significance of group separation.

Principal component analysis (PCoA) is a visualization method used to study similarities or differences in data. It enables the analysis of variations among individuals or groups through the relative positional relationships of sample points. As shown in Figure 4A,E, samples from CR in the developmental stages and maturity stages were distributed in different quadrants from the other samples, indicating significant differences in species composition and proportions in these stages compared to the other samples.

**Figure 5:**
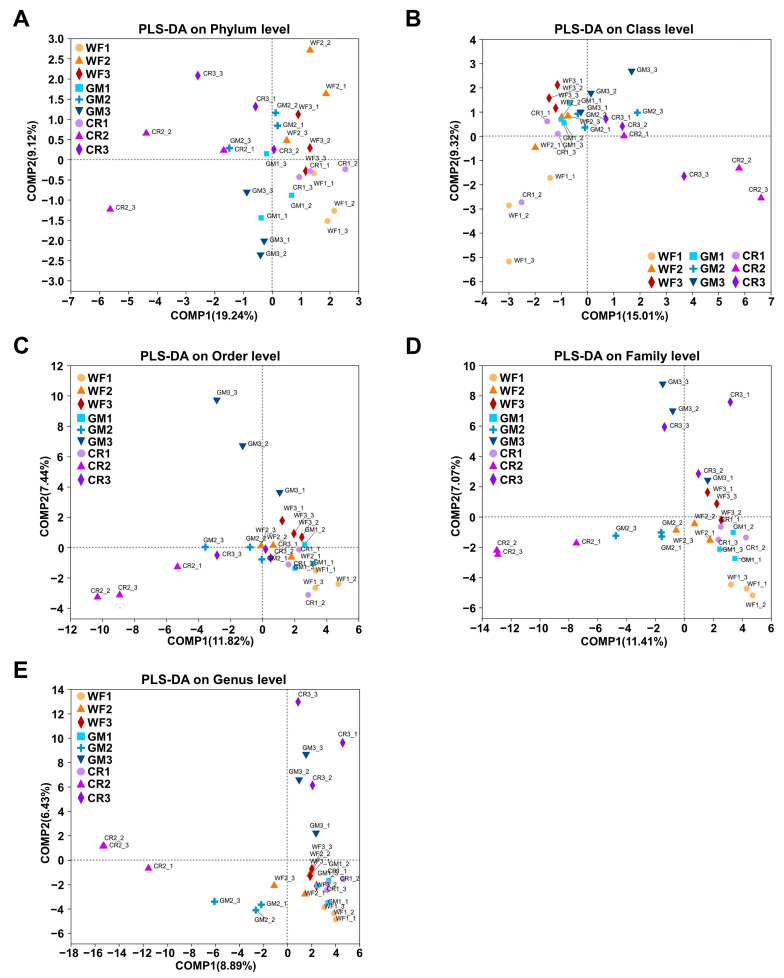
PLS-DA plots visualize β diversity of endophytic bacterial communities across phylum (**A**), class (**B**), order (**C**), family (**D**), and genus (**E**) levels. COMP1 and COMP2 axes represent principal components explaining variance in community composition. Different colors/symbols represent experimental groups.

Partial least squares discriminant analysis (PLS-DA) can maximize differences between groups based on predefined classifications, achieving better separation effects than PCoA. In contrast to PCoA (Figure 5A,E), GM and CR samples from the developmental stages were distributed in the same quadrant, while GM and CR samples from the maturity stages were also distributed in the same quadrant, with the remaining samples distributed in another quadrant. This suggests that GM and CR samples from the developmental stages had higher similarity compared to the other samples, and GM and CR samples from the maturity stages also had higher similarity compared to the others. Overall, both of these groups exhibited greater differences from the rest of the samples.

**Figure 6:**
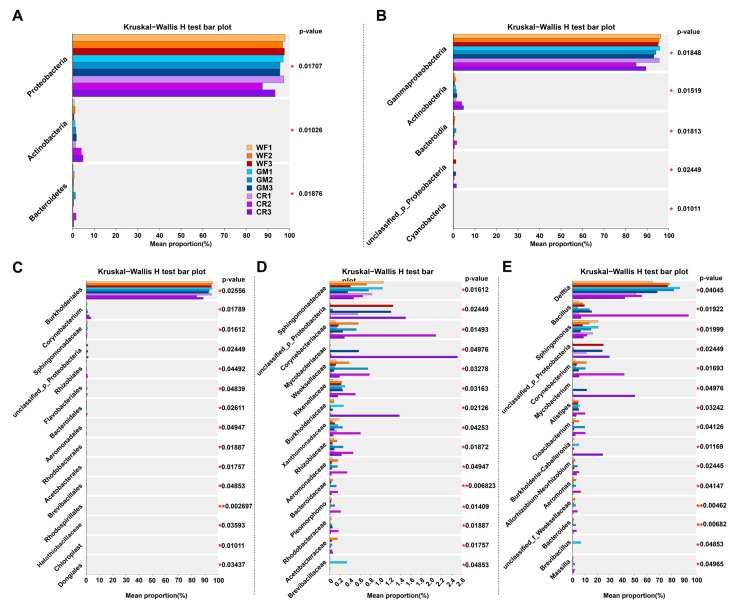
Kruskal–Wallis H test analysis of endophytic bacterial community differences at various taxonomic levels. The figure illustrates the mean proportion of endophytic bacterial communities at phylum (**A**), class (**B**), order (**C**), family (**D**), and genus (**E**) levels across different groups. Horizontal bar charts display the relative abundance of each taxonomic unit. The Kruskal–Wallis H test *p*-values, annotated adjacent to each taxon, indicate the significance of differences between groups. (* indicates significant difference, * *p* < 0.05, and ** *p* < 0.01.).

The Kruskal–Wallis H test is a nonparametric method used to compare the differences between multiple independent samples. The results showed that there was no significant difference in the species of different samples at the phylum, class, and order levels (Figure 6A,C). However, different samples showed significant differences at the family level and the gene level (Figure 6D,E). Overall, the species composition and proportion in the developmental stage and maturity stage were significantly different from those in the other stage.

### 3.5. The LEfSe Multilevel Species Hierarchy Tree Diagram and Latent Dirichlet Assignment (LDA) Showed the Dominant Species Differences in Different Samples

**Figure 7:**
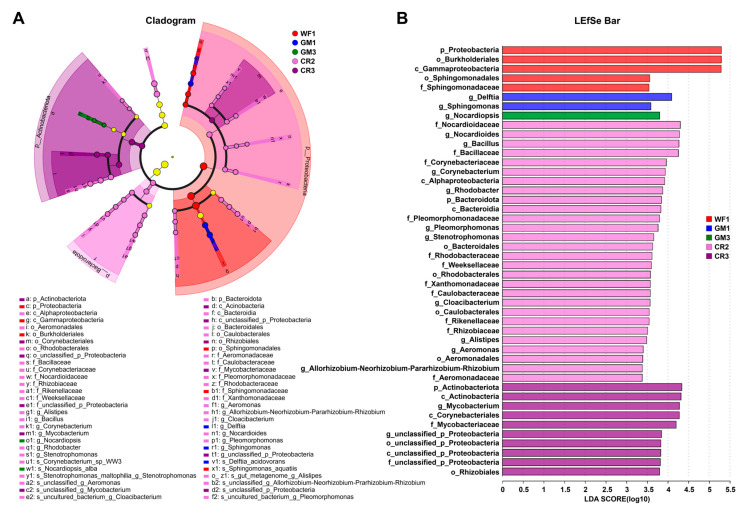
LEfSe multilevel species hierarchy tree diagram and LDA discrimination result diagram. (**A**) LEfSe multilevel species hierarchy tree diagram (cladogram). This cladogram illustrates microbial community structure and discriminative taxa across experimental groups (WF1, GM1, GM3, CR2, CR3). The circular diagram depicts taxonomic hierarchies from phylum to genus/species, with nodes colored by group-specific associations. (**B**) LDA discriminant result graph. The LDA bar plot quantifies microbial taxa with significant differential abundance across the five treatment groups. LDA scores reflect the contribution of species abundance to intergroup divergence, with column colors indicating different experimental groups.

LEfSe and LDA showed that different samples had different dominant species (Figure 7A,B). The results showed that the top five dominant species of WF in the initiation stage samples were *p_Proteobacteria*, *o_Burkholderiales*, *c_Gammaproteobacteria*, *o_Sphingomonadales,* and *f_Sphingomonadaceae*. The dominant species of GM in the initiation stage samples were *g_Delftia* and *g_Sphingomas*. The dominant species of GM in the maturity stage samples were *g_Nocalypsis*. The top five dominant species of CR in the development stage samples were *f_Nocardioidaceae*, *g_Nocardioides*, *g_Bacillus*, *f_Bacillaceae*, and *f_Corynebacteriaceae*. The top five dominant species of CR in the maturity stage samples were *f_Aeromonadaceae*, *p_Actinobacteriota*, *g_Mycobacterium*, *o_Corynebacteriales*, and *f_Mycobacteriaceae*.

### 3.6. Microbial Function Prediction

**Figure 8:**
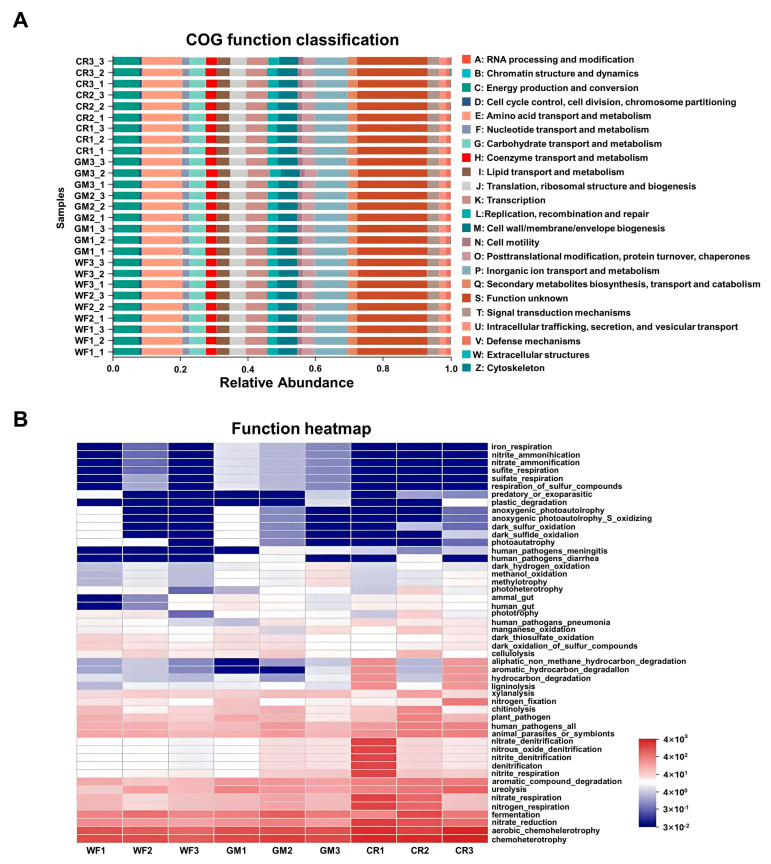
Metabolic functional features of peanut kernel endophytic bacteria. (**A**) Analysis by COG. Different COG groups are displayed in different colors, as listed on the right. (**B**) Analysis by KEGG.

Using PICRUSt10 to standardize the OTU abundance table, we observed differences and changes in the functional genes of microbial communities in metabolic pathways among various samples and aimed to investigate the metabolic functional alterations of peanut kernel endophytic microbiota in response to environmental changes. Through analysis with the Cluster of Orthologous Groups (COG) (Figure 8A) and the Kyoto Encyclopedia of Genes and Genomes (KEGG), compared to WF, CR samples exhibited slight elevations in pathway levels related to cell proliferation and division, such as cell cycle control, cell division, chromosome partitioning, RNA processing and modification, and transcription (Figure 8A,B). This suggests that the metabolic functions of endophytic microbiota in peanut kernels under crop rotation may adapt and adjust to better cope with environmental variations.

### 3.7. Correlation Analysis Between the Endophyte Community of Peanut Pods and the Macronutrient Contents of the Peanut Kernels Under Different Planting Methods

**Figure 9:**
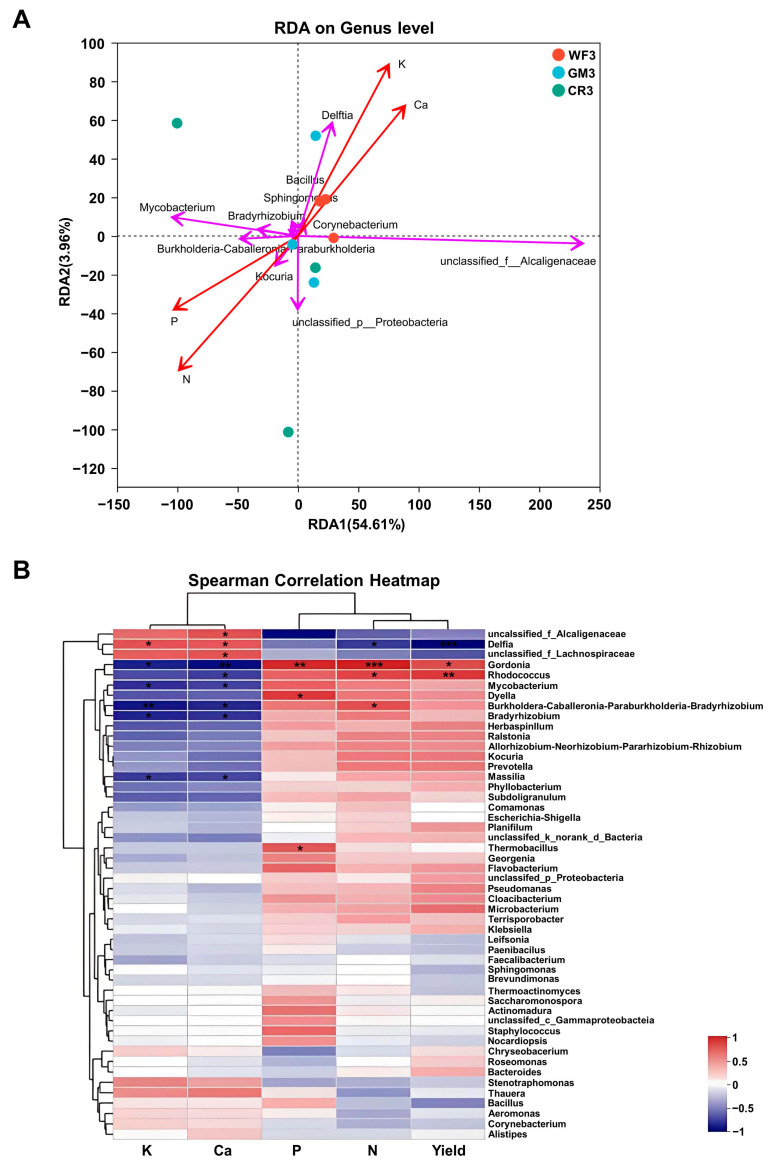
Correlation analysis of peanut kernel endophytic bacterial communities (genus level, >1% abundance) with macroelements (K, Ca, P, N) and yield. (**A**) RDA analysis showing relationships between bacterial community structure, macroelements, and yield. Red arrows: macroelements and yield; purple arrows: bacterial communities. (**B**) Spearman correlation heatmap depicting associations between bacterial communities, macroelements, and yield (* *p* < 0.05, ** *p* < 0.01, *** *p* < 0.001).

In this study, we conducted a correlation analysis between the nitrogen content, phosphorus content, potassium content, calcium content, and structure of endophytic bacterial communities in peanut kernels at the maturity stages, considering these environmental factors across four different tillage systems. The results of RDA (Figure 9A) between the taxonomic classification levels of different samples and the clustering of environmental factors revealed the following: WF showed a negative correlation with K and Ca, while no correlation was observed with N and P; GM exhibited a negative correlation with all environmental factors, with the order of influence being P > Ca > N > K; CR demonstrated a positive correlation with K and Ca but a negative correlation with N and P. These findings suggest that the tillage systems have a significant impact on the relationship between environmental factors and the structure of endophytic bacterial communities in peanut kernels, potentially influencing their ecological functions and interactions.

The Spearman correlation heatmap (Figure 9B) revealed a set of bacterial genera with significant or highly significant relationships between their relative abundances at the genus level and the environmental factors. Notably, *Actinospica*, *Cloacibacterium*, and *Proteiniphilum* exhibited a significant positive correlation with both nitrogen content and phosphorus content. In contrast, *unclassified_f__Alcaligenaceae* showed a significant negative correlation with nitrogen content and phosphorus content, while *Mycobacterium* and *Burkholderia-Caballeronia-Paraburkholderia* had a significant negative correlation with potassium content and calcium content. Additionally, *Allorhizobium-Neorhizobium-Pararhizobium-Rhizobium*, *Bradyrhizobium*, and *unclassified_f__Rhizobiaceae* displayed a significant negative correlation with potassium content. These findings suggest that specific bacterial taxa may play important roles in nutrient cycling and ecosystem functioning in response to varying environmental conditions, and their interactions with environmental factors could have implications for agricultural productivity and sustainability.

## 4. Discussion

### 4.1. Rotation Alleviates Continuous Cropping Obstacles and Increases the Accumulation of Nitrogen in Peanut Kernels and the Yield of Peanut Pods

Continuous cropping for many years not only affects the yield and quality of peanuts but also may have long-term negative impacts on soil ecology [6,24]. Rotation is a basic agricultural practice that has been widely recognized for its role in alleviating harmful effects associated with monocropping or continuous cropping systems [25]. By alternately planting crops, rotation disrupts the cycle of pests and diseases, reduces the accumulation of soil-borne pathogens, and can improve soil structure and fertility [26]. When peanuts are rotated with nonleguminous crops, the subsequent nitrogen-rich soil environment can reduce the need for synthetic fertilizers and improve the nitrogen economy of the entire planting system [27,28]. Studies have demonstrated that crop rotation significantly boosts nitrogen accumulation in peanut kernels, a phenomenon linked to improved nitrogen fixation and enhanced nutrient absorption driven by more diverse soil microbial communities [29]. Our findings further confirm that rotation systems increase peanut pod yield by improving soil quality—a crucial factor not only for immediate productivity but also for sustaining long-term agricultural output. Notably, the nitrogen content in peanut kernels consistently rose, underscoring rotation’s role in optimizing nitrogen uptake, a key element for plant growth that directly influences peanut protein levels and yield. However, while nitrogen availability improved, phosphorus content remained unchanged. This suggests that rotation alone may not universally enhance all nutrient uptake, emphasizing the need for tailored fertilization strategies. In addition, our research also showed that CR and GM systems resulted in a decrease in potassium and calcium contents in peanut kernels. This may be due to the large absorption of potassium and calcium in the soil by the previous crop, and the subsequent application of NPK ternary compound fertilizer is not sufficient to fully satisfy the demand for these elements in peanuts. This finding also suggests that a combination of crop rotation and precision fertilization, in which inputs are adjusted to soil and crop needs, may be the key to maximizing productivity and nutrient quality.

### 4.2. Rotation Improves the Colony Structure of Endophytic Bacteria in Peanut Pods, Increasing the Diversity and Richness of Endophytic Bacteria in Peanut Pods

Recent studies have revealed the close relationship between continuous cropping obstacles and changes in soil microbial communities. In particular, continuous cultivation of the same crop can lead to a relatively large proportion of certain bacterial genera in the soil microbial community. Such bacterial genera may release certain harmful substances or competitively inhibit the growth of other beneficial microorganisms, thereby affecting the normal growth and development of peanuts [30,31]. This imbalance in bacterial communities may be caused by multiple factors, such as soil nutrient imbalance, soil structure damage, and toxic substance accumulation caused by continuous cropping [2]. Our results suggest that GM and CR are effective strategies for alleviating peanut continuous cropping obstacles. This study showed that both GM and CR systems can significantly improve the colony structure of endophytic bacteria in peanut pods. Compared with WF, both GM and CR significantly increased the diversity and abundance of endophytic bacteria in peanut pods, but the effect of CR was more pronounced (Figure 2C,D). This may be because CR improves the soil environment, making the bacterial community structure in the soil more balanced and healthier. Given the critical role of soil microbiota in nutrient acquisition and disease resistance, optimized crop rotation systems promote a more favorable rhizosphere environment, thereby enriching endophytic microbial diversity in peanut pods and supporting sustainable peanut production.

Furthermore, crop rotation can enhance the bioavailability of essential macroelements (e.g., nitrogen, phosphorus, and potassium) in soil through optimized crop sequencing and fertilization strategies. This improvement in nutrient availability stimulates plant growth and subsequently modifies the internal plant environment, creating favorable conditions for endophytic colonization. As symbiotic microorganisms residing within plant tissues, endophytes play crucial roles in enhancing host disease resistance, stress tolerance, and nutrient acquisition [32,33,34]. Therefore, the improvement of the internal environment of plants not only promotes the growth of peanuts but also promotes the abundant reproduction of endophytes, thereby increasing the abundance of endophytes. Our results show that LEfSe and LDA jointly indicate that different samples have different dominant species (Figure 7A,B). At the genus level, the dominant differential genera for GM1 were *Delftia* and *Sphingomonas*, while for GM2, it was *Nocardiopsis*. CR2 had 11 dominant differential genera, with the top three being *Bacillus*, *Nocardioides*, and *Corynebacterium*, while CR3 had two dominant differential genera, namely, *Mycobacterium* and *g__unclassified_p__Proteobacteria*. Previous studies have indicated that *Burkholderiales* is a potential plant pathogen [35,36]. Peanut plants release specific root exudates that can selectively enhance or suppress certain microbial populations. *Burkholderiales* may possess the ability to utilize peanut root exudates more effectively, leading to their proliferation. Studies have shown that rhizosphere-derived *Nocardiopsis alba BH35* can be used as an effective biocontrol agent actinobacterium with antifungal and plant growth-promoting effects [37]. Numerous studies have demonstrated that *Bacillus* has a positive impact on crop growth and soil quality. *Bacillus* promotes plant growth by producing growth-stimulating substances like indole-3-acetic acid (IAA), gibberellins, and cytokinins, which enhance root and shoot development [38]. Additionally, *Bacillus* can induce systemic resistance in plants, activating defense mechanisms that protect plants from disease [39]. *Nocardioides* can exist as mutually beneficial endophytic bacteria in plants [40]. In summary, both GM and CR systems significantly promoted the increase in certain beneficial bacterial groups in peanut pods, suppressed the growth of harmful microorganisms, and provided a healthy soil ecological environment for peanuts.

### 4.3. The Endophytic Bacteria in Peanut Pods Have Two Possible Sources: Vertical Transmission and the Colonization of Soil Rhizosphere Bacteria Within the Pods

The abundance and diversity of endophytic bacteria in peanut pods tend to gradually increase as pods develop. Similar dynamic changes in endophytic bacteria were observed under the three treatment methods of WF, GM, and CR. Regardless of the treatment, the diversity and abundance of endophytic bacteria in peanut pods reached their highest levels during the mature stage, followed by the developmental stage, and were lowest during the initiation stage. This observation sheds light on the possible sources of endophytic bacteria in peanut pods. Apparently, a portion of the endophytic bacteria is inherited from the parent plant, but as time passes and the pods develop, an increasing number of endophytic bacteria begin to colonize. This suggests that the soil microbial community is an important source of endophytic bacteria [41]. Microorganisms from the soil can enter plant tissues through various means and grow and reproduce within them, thus becoming a part of the plant’s endophytic bacteria. Our results reveal a potentially close connection between soil microbial communities and plant endophytic bacteria, demonstrating the direct impact of soil microorganisms on the diversity and abundance of endophytic bacteria in peanut pods. Therefore, optimizing soil microbial communities is an important approach to increasing the diversity and abundance of endophytic bacteria in peanuts or other crops. By improving the cultivation pattern, applying fertilizers reasonably, and planting green manure crops, we can improve the soil environment, providing more favorable conditions for endophytic bacterial colonization in peanut pods, thereby promoting peanut growth and increasing yield.

## 5. Conclusions

This study demonstrates that GM and CR systems effectively alleviate continuous cropping obstacles in peanut cultivation by significantly increasing pod yield and enhancing nitrogen accumulation in kernels. The findings also highlight the strong influence of GM and CR on reshaping the endophytic bacterial community in peanut pods. Both the ACE and Shannon indices increased, improving the colony structure of endophytic bacteria in peanut pods. CR specifically enriched nitrogen-fixing and growth-promoting genera *(Bacillus*, *Rhodobacter*, and *Allorhizobium*-*Neorhizobium*-*Pararhizobium*-*Rhizobium*), while GM boosted stress-tolerant bacteria (*Delftia*, *Sphingomonas*), with microbial diversity peaking during pod development stages. Based on the current findings, we recommend that CR with alternating GM succession during peanut cultivation improve soil nitrogen cycling, microbial diversity, and peanut yields while reducing reliance on chemical inputs. Further experiments could focus on optimizing the rotation period to maximize the synergistic effects on endophytic microbiome regulation and peanut productivity.

## Figures and Tables

**Table 1:** Macroelements of peanut kernels and peanut (*Arachis hypogaea* L.) pod yield in different cropping patterns.

Cropping Patterns	Nitrogen Content (g/kg)	Phosphorus Content (g/kg)	Potassium Content (g/kg)	Calcium Content (g/kg)	Peanut Pod Yield (t/ha)
WF	40.39 ± 0.25 c	2.17 ± 0.07 a	6.70 ± 0.06 a	7.68 ± 0.09 a	2.71 ± 0.08 b
GM	40.99 ± 0.07 b	2.32 ± 0.09 a	6.21 ± 0.15 b	7.13 ± 0.19 b	2.58 ± 0.50 b
CR	43.14 ± 0.08 a	2.32 ±0.11 a	5.44 ± 0.25 c	6.49 ± 0.13 c	3.60 ± 0.20 a

Data are the means ± S.D., *n* = 3. The different lowercase in the same column indicates significant difference between different cropping patterns (*p* < 0.05, one-way ANOVA). WF: winter fallow; GM: green manure; CR: crop rotation.

## Data Availability

The datasets generated during the current study have been submitted to National Center for Biotechnology Information (NCBI) Sequence Read Archine (SRA) database number: PRJNA1098361.
